# Haematopoietic Stem Cell Transplantation for Primary Haemophagocytic Lymphohistiocytosis

**DOI:** 10.3389/fped.2019.00435

**Published:** 2019-10-25

**Authors:** Kai Lehmberg, Despina Moshous, Claire Booth

**Affiliations:** ^1^Division of Paediatric Stem Cell Transplantation and Immunology, University Medical Centre Hamburg Eppendorf, Hamburg, Germany; ^2^Department of Immunohematology, Necker-Enfants Malades Hospital, APHP, and Imagine Institute, Inserm U 1163, Descartes University, Paris Sorbonne Cité, Paris, France; ^3^Department of Paediatric Immunology, Great Ormond Street Hospital, London, United Kingdom; ^4^Molecular and Cellular Immunology Section, UCL Great Ormond Street Institute of Child Health, London, United Kingdom

**Keywords:** haemophagocytic lymhohistiocytosis, macrophage activation syndrome, haematopoietic stem cell transplantation, gene therapy, veno-occlusive disease, mixed chimerism, reduced toxicity conditioning

## Abstract

Haematopoietic stem cell transplantation currently remains the only curative treatment of primary forms of haemophagocytic lymphohistiocytosis (HLH). Rapid diagnosis, efficient primary treatment of hyperinflammation, and conditioning regimens tailored to this demanding condition have substantially improved prognosis in the past 40 years. However, refractory hyperinflammation, central nervous system (CNS) involvement, unavailability of matched donors, susceptibility to conditioning-related toxicities, and a high frequency of mixed chimaerism remain a challenge in a substantial proportion of patients. Gene therapeutic approaches for several genetic defects of primary HLH are being developed at pre-clinical and translational levels.

## Key Messages

- Remission at HSCT is a key factor of survival in HLH patients. To avoid reactivations, time to transplant should be kept at a minimum, which may require alternative donors.
- The high prevalence of fatal veno-occlusive disease after full myeloablative busulfan-based conditioning in HLH patients has been substantially curtailed with conditioning regimens of reduced toxicity.
- Achieving high levels of sustainable donor chimerism in the absence of GvHD remains a challenge in HLH patients when using RIC regimens.

## Introduction

Haemophagocytic lymphohistiocytosis (HLH) is a hyperinflammatory condition that may occur in a primary or secondary form. The clinical picture is characterized by the triad of fever, splenomegaly, and symptoms of cytopenia. In combination with laboratory and cytological findings, they form a set of defining criteria (HLH-2004). Untreated full scale primary HLH is usually fatal (1).

Genetic defects predisposing to primary HLH are found in autosomal recessive familial HLH (FHL) 2-5, Griscelli syndrome type 2 (GS2), Chediak-Higashi syndrome (CHS), X-linked lymphoproliferative disease type 1 (XLP1), and X-linked inhibitor of apoptosis (XIAP) deficiency. In primary HLH, defective cytotoxic function of CD8+ T cells and NK cells lead to excessive secretion of numerous inflammatory cytokines that can be made responsible for different clinical features of HLH (2). However, other immunodeficiencies (e.g., chronic granulomatous disease) display a predisposition to HLH as well (3). Pathogenesis of secondary HLH, associated with infectious, malignant, autoimmune, autoinflammatory, or metabolic diseases, is less well-elucidated.

Taking into account the rarity of primary HLH and the retrospective nature of the majority of published studies on HSCT in this condition, data summarized in this review must be interpreted with caution.

## Pre-HSCT Treatment

Treatment of HLH aims at decreasing inflammation and treating rigorously the underlying trigger, if any. The principal goal of induction therapy is to suppress the life-threatening inflammatory process. Once remission of HLH is achieved, patients with primary HLH require allogeneic haematopoietic stem cell transplantation (HSCT), the only curative therapy to date preventing relapses and central nervous system (CNS) disease progression. Isolated CNS-HLH remains a challenge for diagnosis and treatment. The quality of remission prior to HSCT is an important survival factor, especially in patients who lack HLA identical donors (4, 5). Despite significant treatment progress, mortality remains high. The etoposide-based treatment induction in the HLH-94 (dexamethasone, etoposide, intrathecal methotrexate) and HLH-2004 studies (addition of cyclosporine A upfront) resulted in 20–30% mortality before HSCT and 5-year overall survival (including HSCT) of ~60% (6, 7). Etoposide-based regimens may be associated with toxicities contributing to pre HSCT mortality, including bone marrow suppression and infection, as well as secondary malignancy.

A better understanding of the pathophysiology of primary HLH has opened new avenues for targeted immunotherapy. Based on previous observations showing the efficacy of immunotherapy directed selectively against T cells by using anti-thymocyte globulin (ATG) in FHL patients (8), the anti-CD52 antibody alemtuzumab has been used efficaciously in refractory HLH (9). A prospective trial with alemtuzumab as first line treatment in primary HLH is currently ongoing (NCT02472054).

New treatment options are emerging in cytokine-driven immune disorders. In fact, many of the elevated cytokines in HLH signal via Janus Kinase (JAK) and Signal Transducer and Activator of Transcription (STAT)-associated receptors, therefore JAK1/2 have become targets for therapeutic intervention. Ruxolitinib, a JAK1/2, inhibitor has shown efficacy in mouse models of HLH (10, 11); it has also been used in humans in isolated cases of refractory primary and secondary HLH (12–14). The role of interferon (IFN)-γ in the development of HLH has led to the development of the anti-IFN-γ monoclonal antibody emapalumab which proved efficient in mice (15, 16), clinical studies in HLH patients are ongoing (NCT03312751).

## Conditioning Regimens and Adverse Events

HSCT with myeloablative conditioning regimens containing busulfan, cyclophosphamide, and VP16 resulted in substantial transplantation related mortality (overall survival 43–64%), in particular related to veno-occlusive disease (VOD) (4, 5, 17, 18). The advent of conditioning regimens with reduced toxicity based on melphalan as alkylating agent in combination with fludarabine led to a substantial improvement of survival with a favorable toxicity profile and a very low rate of lethal VOD (18–20). Treosulfan-based schemes with thiotepa rendered similar results (21). Mostly retrospectively determined survival rates in patients with HLH have been reported at 44–100% (21–25) for treosulfan-based protocols and 51–92% (18, 23, 26–28) for melphalan-based regimens (Table 1). Whether sub-myeloablative busulfan will constitute an alternative approach remains to be demonstrated.

**Table 1:** Compilation of selected publications on HSCT for HLH after conditioning regimens with reduced toxicity.

	***n***	**Conditioning regimen**	**Overall survival [%]**	**Low donor chimaerism or any intervention [%]**	**2 HSCT [%]**	**aGvHD [%]**	**cGvHD [%]**
Cooper 2006/8 (19, 28)	25	Melphalan, fludarabine, alemtuzumab	84	26	0	33 (3°-4°)	25
Marsh 2010 (25)	26	Melphalan, fludarabin, alemtuzumab	92 (3 yrs)	53	4	27 (2°-3°)	12
Lehmberg 2013 (21)	19	Treosulfan, (thiotepa), fludarabine, alemtuzumab	100	42	11	5 (3°-4°)	0
Slatter 2018 (22)	16	Treosulfan, fludarabine, alemtuzumab	44	n.a.	n.a.	n.a.	n.a.
Allen 2018 (26)	34	Melphalan, fludarabine, alemtuzumab	67 (1.5 yrs)	40	n.a.	17 (3°-4°)	27
Wustrau 2019 (29)	60	Treosulfan or melphalan, (thiotepa), fludarabine alemtuzumab or ATG	75 (5 yrs)	42	15	13 (3°-4°)	5

*n.a., not available*.

It is challenging to tailor conditioning and serotherapy achieving high levels of donor chimerism in the absence of graft-versus-host disease (GvHD), particularly in mismatched donors (21). A donor chimerism of ≥20–30% has been shown to protect against disease reactivation; and even lower rates do not inevitably result in recurrence of HLH (30). However, the dynamics of incipient appearance of recipient cells is poorly predictable. Studies and case series using treosulfan or melphalan in HLH patients report 30–100% of patients with mixed chimerism and frequent necessity for secondary cellular cell therapy, including donor lymphocyte infusions and secondary HSCT (18, 22, 26, 27).

A prospective study reported a chronic GvHD rate of 27% (26). Serotherapy has a dual role in HLH patients, as it not only prevents GvHD and rejection but equally dampens residual disease activity. ATG and alemtuzumab have been reported as lymphodepleting agents in larger series. In a retrospective analysis of 60 patients, no significant difference regarding the occurrence of mixed chimerism between both agents could be detected (29). A “distal” administration of serotherapy reduces the risk of mixed chimerism, however leads to an elevated rate of graft-versus-host disease (GvHD) (31).

## Alternative Donors

In primary HLH, allogeneic hematopoietic stem cell transplantation is the only curative treatment option and should ideally be offered as soon as remission from the activation syndrome is achieved. Especially in patients lacking HLA identical donors, the quality of remission is essential to improve survival rates (4, 5). Alternative donors should therefore be considered early. Unrelated umbilical cord blood transplantation may provide a readily available alternative donor source, and 6-year overall survival of 55% has been reported in a recent analysis of 118 patients receiving mostly busulfan-based conditioning (32).

Haploidentical parental donors provide the opportunity for virtually all patients to benefit from HSCT. However, T-lymphocyte depleted haplo-identical transplantation is associated with high risk of graft rejection when omitting cyclophosphamide and using busulfan/fludarabine or treosulfan/fludarabine in the conditioning regimen as currently recommended in the EBMT/ESID guidelines (33). T-lymphocyte replete HSCT with administration of cyclophosphamide post transplantation in order to reduce the incidence of GvHD while sparing other T-lymphocytes seems to be a valid option in primary HLH patients who lack a conventional donor. In a small series of 10 patients with primary HLH, treatment related mortality was 20% with one additional death in a patient who died from central line sepsis on parenteral nutrition 9 months post HSCT, 6 out these 10 patients have been already reported (34). The possibility of further reducing the conditioning intensity to decrease toxicity needs to be assessed.

## Special Considerations

Although HSCT is a recognized curative treatment for primary HLH, certain conditions can prove more challenging, particularly when deciding if, or when, to proceed with transplant. It is well-established that patients entering HSCT with active disease have a worse outcome, and pre-emptive transplant is generally advocated in patients with proven genetic lesions and HLH associated with immune deficiency (e.g., XLP). Overall survival of second affected children in families with primary HLH was reported to be 93%, as compared to 64% in their index siblings (35).

HLH conditions associated with pigmentary disorders have variable risks of HLH and often significant neurological involvement. Patients with Griscelli syndrome (GS2) are at high risk of developing HLH but results of HSCT are favorable (36–38). Most series of GS2 patients report 40–50% significant neurological sequelae mostly related to CNS HLH (36) and families must be counseled appropriately. The risks of developing HLH are lower in Chediak-Higashi syndrome where decision to transplant should be based on genetic and cytotoxic function (39) and lower still in Hermansky-Pudlak syndrome type 2 where pre-emptive HSCT is not justified (40).

XIAP deficient patients can be particularly difficult to treat. HSCT related mortality in this patient population is worse than patients undergoing HSCT for other forms of familial HLH, even when using a reduced intensity conditioning regime (57% probability of survival at 1 year and 43% probability of long-term survival) (41). These patients may develop significant GvHD that can be resistant to therapy, contributing to morbidity and mortality (42). Interestingly a case series from Japan reported a 90% survival but 50% of patients developed inflammatory episodes post-HSCT. A high proportion of patients will manifest inflammatory enteropathy as part of their disease, and this can be slow to resolve post-HSCT.

Some patients with FHL5 due to mutations in *STXBP2*, may present severe, osmotic diarrhea, and renal proximal tubular dysfunction. These clinical manifestations are related to defective membrane trafficking in the gut and kidney and persist also after successful HSCT (43–45).

Although CNS involvement is present in around 30% of cases of systemic HLH (7), isolated CNS HLH is becoming increasingly recognized. Diagnosis and management can be challenging, and specialist advice must be sought early (46). Early intervention with CNS directed therapy appears key. HSCT is curative in these cases, but cannot revert manifest CNS damage (47).

## Gene Therapy for HLH

Autologous HSC gene therapy (GT) has been developed for several primary immunodeficiencies and offers an efficacious alternative treatment option for patients lacking a suitable donor for HSCT (see review on “Autologous stem cell-based gene therapy for inherited disorders: state-of-the-art and future prospects”). Preclinical and translational studies of HSC GT are now underway for several forms of primary HLH including FHL2, FHL3, XLP, and XIAP deficiency. In addition, proof of concept for adoptive transfer of autologous gene corrected T-lymphocytes has now been published in three of these conditions (48); a therapeutic strategy, which may act as a bridge to transplant or perhaps offer longer-term clinical benefit.

Lentiviral transfer of the perforin gene into haematopoietic progenitors in a *Prf* ^−/−^ murine model led to the recovery of NK cell function, CD8 T-lymphocyte cytotoxicity and reduced IFN-ɤ secretion *in vitro* alongside improvement in cytopaenias and cytokine hypersecretion when gene corrected mice were challenged with lymphocytic choriomeningitis virus (LCMV) to induce an HLH phenotype (49). Subsequently, this group also demonstrated that gene correction of T-lymphocytes reduced disease activity and prevented HLH *in vivo* alongside restoring cytotoxicity in patient T-lymphocytes (48). Soheili et al. demonstrated that both an HSC and T-lymphocyte approach can ameliorate defects in FHL3 with again, reduction of HLH symptoms and biomarkers in an LCMV challenged Jinx mouse model following transfer of gene modified HSCs (50) and rescue of cytotoxicity in patient T-lymphocytes in a tumor model and degranulation assay (51). Dettmer et al. showed it was possible to efficiently transduce hyperactivated patients T-lymphocytes with a retroviral vector containing the *UNC13D* gene generating functionally corrected cells (52).

Preclinical studies have demonstrated proof of concept for HSC and T-lymphocyte gene therapy strategies for XLP with correction of immune abnormalities using both approaches. *Sap*^−/y^ mice reconstituted with lentiviral mediated HSC gene correction showed recovery of NK cell cytotoxicity and humoral defects including immunoglobulin levels and T-lymphocyte dependent antigen responses (53). Transfer of gene corrected T-lymphocytes in *Sap*^−/y^ mice restored T-lymphocyte dependent antigen responses and germinal center formation whilst gene transfer in patient T-lymphocytes led to functional recovery of T follicular helper lymphocytes and cytotoxicity (both *in vitro* and *in vivo* using a xenograft lymphoma model) (54). Preclinical studies are underway investigating HSC gene therapy as a possible therapeutic option for XIAP deficiency (unpublished data).

## Conclusions and Outlook

Despite significant progress, front-line treatment and HSCT procedures still require improvement to further reduce mortality and long-term sequelae of this potentially devastating condition. New therapeutic agents may complement current standards of care, optimization of conditioning regimens may overcome remaining challenges, and gene therapy approaches will probably be available in the future for the most frequent HLH defects.

## Author Contributions

All authors wrote the review and approved of the final version.

### Conflict of Interest

KL is member of an advisory board of SOBI. CB has received consulting fees from SOBI and Novimmune. The remaining author declares that the research was conducted in the absence of any commercial or financial relationships that could be construed as a potential conflict of interest. The handling editor AG declared a current collaboration with the author CB.

## Abbreviations

ATG: anti-thymocyte globulin
CHS: Chediak Higashi syndrome
CNS: central nervous system
EBMT: European Society of Bone Marrow Transplantation
ESID: European Society of Immunodeficiencies
FHL: familial HLH
GS2: Griscelli syndrome type 2
GT: gene therapy
GvHD: graft-versus-host disease
HLH: haemophagocytic lymphohistiocytosis
HSCT: haematopoietic stem cell transplantation
IFN: interferon
JAK: janus kinase
LCMV: lymphocytic choriomeningitis virus
STAT: Signal Transducer and Activator of Transcription
VOD: veno-occlusive disease
XIAP: x-linked inhibitor of apoptosis
XLP: x-linked lymphoproliferative disease.
